# Effects of home delivery on colostrum avoidance practices in North Wollo zone, an urban setting, Ethiopia: a cross sectional study

**DOI:** 10.1186/s41043-018-0134-4

**Published:** 2018-02-26

**Authors:** Nigus Bililign Yimer, Misgan Legesse Liben

**Affiliations:** 1 Department of Midwifery, Faculty of Health Sciences, Woldia University, Amhara, Ethiopia; 2 Department of Public Health, College of Medical and Health sciences, Samara University, P.O.Box 132, Afar, Ethiopia

**Keywords:** Colostrum, Home delivery, Kobo, Lalibela, Woldia, North Wollo, Ethiopia

## Abstract

**Background:**

Colostrum is the first liquid that is produced in the first few days after delivery. It is the perfect first food for newborns which is considered as an infant’s first immunization. Despite of this fact colostrum is discarded as unclean and bad for the infant’s health. This study aimed to investigate the prevalence and the factors associated with colostrum avoidance in Woldia, Kobo and Lalibela town administrations of North Wollo zone.

**Methods:**

A quantitative community based cross sectional study was employed in March 2015 on 810 mothers of children aged less than 24 months. Descriptive statistics, binary and multivariable logistic regression analysis were employed to identify the factors associated with colostrum avoidance. Variables with a *p*-value < 0.05 in the multivariable model were identified as predictors of colostrum avoidance practices.

**Results:**

Colostrum was discarded by 12.0% (95%CI: 10.0–14.0%) of mothers of children aged less than 24 months. In multivariable logistic regression analysis late initiation of breastfeeding [AOR (95% CI) =2.03 (1.18, 3.49)], prelacteal feeding [AOR (95% CI) =3.38 (1.83, 6.24)], mothers not living with their husband [AOR (95% CI) = 2.24 (1.22, 4.12)] and delivering the index child at home [AOR (95% CI) =2.92 (1.521, 5.59)] were independent positive predictors of colostrum avoidance practices.

**Conclusion:**

The foundation of any nutrition package for the prevention of childhood malnutrition is the promotion of an optimal breastfeeding practices, including colostrum feeding, in the community. Therefore, promoting institutional delivery, early initiation of breastfeeding and creating awareness on the dangers of prelacteal feeding and the advantages of colostrum feeding are recommended interventions to reduce colostrum avoidance practices in the study area.

## Background

World Health Organization (WHO) and united nation international children’s emergency fund (UNICEF) recommend exclusive breastfeeding for children up to 6 months of age, and to nourish them with appropriate complementary foods and continued breastfeeding from 6 months until 2 years or beyond [1]. Ethiopia also adopts this recommendation [2, 3]. Exclusive breastfeeding is an infant’s breast milk consumption without supplementation of any type of foods or drinks, except for vitamins, minerals and necessary medications up to the age of 6 months [1, 2, 4].

Colostrum is the first liquid that is produced in the first few days after delivery [4]. Colostrum flows in very small amounts that suit the infant’s very small stomach and the immature kidneys that cannot handle large amount of fluid. Compared to matured milk, colostrum is slightly yellow, more viscous, and thicker. It contains growth factors and protective proteins (immune factors) as well as all other nutrients the newborn needs to survive. Colostrum is an infants’ first immunization against many bacteria and viruses. Colostrum is also a laxative which helps the baby to pass meconium (the first sticky black stool). Colostrum is therefore the perfect first food for newborns [1]. Despite of this fact colostrum is discarded as unclean and bad for the infant’s health [5–7].

Colostrum avoidance is failure to feed infants with the first, thick and yellowish milk that is produced in the first few days after birth [1]. It has a significant association with increased odds of malnutrition among children aged less than 5 years. In India infants who fed on colostrum were less likely to be stunted and wasted compared to children who were deprived of colostrum [8]. In West Gojjam children deprived of colostrum were more likely to be stunted compared to children who had fed on colostrum [9]. Children who fed on colostrum were less likely to be malnourished compared to children who deprived of colostrum in Nigeria [10]. Colostrum avoidance has also a negative association with optimal breastfeeding practices [6, 11, 12].

Prelacteal feeding is a common practice in some cultures over colostrum feeding [7, 13–18]. Prelacteal feeds are foods and/or drinks other than human milk, given to newborns before breastfeeding initiation usually on the first 3 days of life. These traditional feedings restrict suckling, making breastfeeding more challenging to establish [1, 4, 19]. Hence, assessing the factors associated with colostrum avoidance is essential in maintaining optimal breastfeeding practices. In addition limited studies are conducted in Ethiopia to describe colostrum avoidance practices. This study aimed to investigate the prevalence and factors associated with colostrum avoidance in Woldia, Kobo and Lalibela town administrations of North Wollo zone.

## Methods

### Study setting and participants

North Wollo zone is one of the ten zones (the smallest administrative units next to region in Ethiopia) of the Amhara Regional State. It is bordered on the south by South Wollo Zone, on the west by South Gondar, on the north by Wag Hemra, on the northeast by Tigray Region, and on the east by Afar Region. The towns in North Wollo zone include Lalibela, Woldia and Kobo. Based on the 2007 Census conducted by the central statistical agency of Ethiopia, this Zone has a total population of 1,500,303, of whom 752,895 are men and 747,408 women. The largest ethnic group reported in North Wollo zone is the Amhara (99.38%) and Amharic is spoken as a first language by 99.28%.

This study was conducted in Woldia, Kobo and Lalibela towns of North Wollo zone in March 2015. Woldia is the capital of North Wollo zone. It is located 521 km north of Addis Ababa and about 360 km south of the capital of Amhara regional state, Bahirdar. This town has eight kebeles (the smallest administrative units next to district in Ethiopia). Based on the 2007 national census conducted by the central statistical agency of Ethiopia, this town has a total population of 46,139, of whom 23,000 are men and 23,139 women. Kobo town is located in the northeast corner of North Wollo zone. Kobo and Lalibela town administrations consist of five and two kebeles, respectively. The majority of the inhabitants in the three towns (Woldia, Kobo and Lalibela) were Amhara and followers of Ethiopian orthodox Christians.

A quantitative community based cross sectional study was employed to survey 810 mothers of children aged less than 24 months. The sample size was determined using a single population proportion formula:

$$ \mathrm{n}=\mathrm{D}\left[\frac{{\left(\mathrm{Z}\frac{\upalpha}{2}\right)}^2\mathrm{p}\left(1-\mathrm{p}\right)}{{\mathrm{d}}^2}\right] $$

Where n = required sample size, Z = critical value for normal distribution at 95% confidence level (1.96), P = prevalence of colostrum avoidance (39.8%) [20], d = 0.05 (5% margin of error), D = 2 (design effect), and an estimated non-response rate of 10%.

### Sampling procedure and technique

First the three towns were selected purposely since these towns are the first three top ranked town administrations (Woldia, Kobo and Lalibela) in North Wollo Zone. Out of the fifteen kebeles in the three towns, eight kebeles were randomly selected. Presurvey was done before the actual period of data collection to know which households have the targeted mother-child pairs. As a result, there were 6013 households having the targeted mother-child pairs in the selected eight Kebeles. Then, the sample size was proportionally allocated to each selected kebeles. At the time of survey, from each household unit one eligible mother who had a biological child aged less than 24 months was selected. If there were more than one mother with children aged less than 24 months in one household unit, one mother with the youngest child was selected. From mothers who had two children aged less than 24 months, the youngest child was selected. If mothers had twin children aged less than 24 months, one child was selected by lottery. Non-biological and mothers who are unable to communicate were excluded from the study.

### Study variables

In this study the outcome variable was colostrum avoidance among mothers of children aged less than 24 months. Colostrum avoidance is failure to feed infants with the first, thick and yellowish milk that is produced in the first 3 days after birth [1]. Discarding colostrum was coded as “1” while colostrum feeding was coded as “0” for regression analysis. The independent variables were maternal characteristics (age, parity, educational status, religion, ethnicity, marital status, occupation), maternal health service and obstetric variables (antenatal care visit, place of delivery, mode of delivery, postnatal care visit), child’s sex, breastfeeding initiation, breastfeeding counseling during antenatal care visits and prelacteal feeding. Prelacteal feeding was understood as providing foods and/or drinks other than human milk for the infant before breastfeeding initiation [1].

### Data collection procedure and quality control

Data were collected using a pre-tested, structured and interviewer administered questionnaire. The questionnaire was prepared first in English and translated to Amharic (local language), then back to English to check for consistency. The Amharic version of the questionnaire was used to collect the data. The data were collected by six diploma midwives. The data collectors and the supervisors (three BSc nurses) were trained for 2 days by the investigators on the study instrument, consent form, how to interview and data collection procedures.

Then questionnaire was pretested on mother-child pairs in two kebeles which were not included in the research. The pretest was done to ensure clarity, wordings, logical sequence and skip patterns of the questions. Then the pretest amendments on the questionnaire were made accordingly. The supervisors had checked the day to day activity of data collectors regarding the completion of questionnaires, clarity of responses and proper coding of the responses.

### Data processing and analysis

The data were checked for completeness and inconsistencies. It was also cleaned, coded and entered into EpiData version 3.02, then exported to the SPSS 20.0 statistical package for analysis. Descriptive statistics were used to show the prevalence of colostrum avoidance practices and socio-demographic characteristics.

Binary logistic regression analysis was performed. The crude odds ratio (COR) with 95% confidence interval was estimated to assess the association between each independent variables and the outcome variable, and to select candidate variables for the multivariable logistic regression analysis. Variables found statistically significant at *p*-value < 0.25 [21] during binary logistic regression analysis were included in the multivariable logistic regression model.

The Hosmer-Lemeshow goodness-of-fit with enter procedure was used to test for model fitness. Adjusted odds ratio (AOR) with 95% confidence interval was estimated to assess the strength of the association, and a *p*-value < 0.05 was used to declare the statistical significance in the multivariable analysis. Variables with *p*-value < 0.05 in the multivariable logistic regression analysis were considered as significant and independent predictors of colostrum avoidance.

### Ethical consideration

The study was approved by institutional research ethics review committee (IRERC) of Woldia University. An official letter was written from research and development office of Woldia University to Woldia, Kobo and Lalibela town’s administration office. Then permission and support letter was written to each selected kebeles. Informed verbal consent was taken from the participants before the interview. The participants were also assured about the confidentiality of the information they provided.

## Results

### Socio-demographic characteristics of the study participants

A total of 782 mother-child pairs were included in the study, yielding a response rate of 96.5%. The mean (±SD) age of mothers was 27.03 (±SD 5.48) years and ranged from 15 to 48 years. Majority of the mothers (72.9%) had attended formal education and 76.4% were in the age group of 20–34 years (Table 1).

**Table 1:** Socio-demographic characteristics of mothers of children aged less than 24 months (*n* = 782) in North Wollo zone, Northeastern Ethiopia, 2015

Variables	Frequency (n)	Percent (%)
Age of mother		
< 20	88	11.3
20–34	598	76.4
> 34	96	12.3
Educational status		
No formal education	212	27.1
Formal education	570	72.9
Marital status		
Single	31	4.0
Married	681	87.1
Divorced	66	8.4
Widowed	4	0.5
Religion		
Orthodox	662	84.7
Muslim	109	13.9
Protestant	10	1.4
Ethnicity		
Amhara	755	96.6
Tigray	23	2.9
Guraghe	4	0.5
Parity		
1	362	46.3
2–3	329	42.1
≥ 4	91	11.6
Maternal occupation		
House wife	473	60.5
Merchant	112	14.3
Government Employee	101	13.0
Daily laborer	52	6.7
Student	43	5.5
Sex of the index child		
Male	413	52.8
Female	369	47.2

### Maternal health service utilization

Ninety four percent of mothers had attended antenatal care visits. Of these only 41.6% were counseled about breastfeeding. About 90% of respondents delivered in health institution. Nearly 54% of mothers attended postnatal care visits (Table 2).

**Table 2:** Distribution of mothers based on maternal health service utilization (*n* = 782) in North Wollo zone, Northeastern Ethiopia, 2015

Variables	Frequency (n)	Percent (%)
Antenatal care (ANC) checkup^a^		
Yes	735	94.0
No	47	6.0
Counseling on breastfeeding during ANC checkup		
Yes	325	44.2
No	410	55.8
Number of ANC checkup		
1	14	1.9
2–3	218	29.7
≥ 4	503	68.4
Place of delivery		
Health institution	700	89.5
Home	82	10.5
Mode of delivery		
Vaginal delivery	655	83.8
Cesarean section	127	16.2
Postnatal care visit		
Yes	420	53.7
No	362	46.3

^a^indicates at least one ANC visit

### Breast feeding practices

About 98 % (98.3%) of mothers had ever breastfed their index child. Of those who had ever breastfed, 601(78.2%) mothers initiated breast feeding within 1 h of birth. Colostrum was discarded by 12.0% (95% CI: 10.0–14.0%) of mothers of children aged less than 24 months (Table 3).

**Table 3:** Breastfeeding patterns (*n* = 782) in North Wollo zone, Northeastern Ethiopia, 2015

Variables	Frequency (n)	Percent (%)
Ever breastfeeding		
Yes	769	98.3
No	13	1.7
Early initiation of breastfeeding		
Yes	601	78.2
No	168	21.8
Prelacteal feeding		
Yes	83	10.8
No	686	89.2
Colostrum feeding		
Yes	688	88.0
No	94	12.0
Maternal awareness on advantages of colostrum		
Yes	504	64.5
No	278	35.5

### Factors associated with colostrum avoidance

Binary logistic regression showed that breastfeeding initiation time, attending antenatal care visits, prelacteal feeding, marital status, parity and place of delivery were associated with colostrum avoidance.

In multivariable logistic regression analysis late initiation of breastfeeding, prelacteal feeding, mothers who live without their husband and delivering the index child at home remained significant as independent positive predictors of colostrum avoidance among mothers of children aged less than 24 months. Mothers who initiated breastfeeding after 1 h of delivery [AOR (95% CI) =2.03 (1.18, 3.49)] were more likely to discard colostrum compared to mothers who initiated breastfeeding within 1 h after delivery. Mothers who practiced prelacteal feeding [AOR (95% CI) =3.38 (1.83, 6.24)] were more likely to avoid colostrum compared to mothers who avoid prelacteal feeds. Compared to mothers who live with their husband, mothers who live without their husband were more likely to discard colostrum [AOR (95% CI) = 2.24 (1.22, 4.11)]. Mothers who delivered the index child at home were 2.9 times [AOR (95% CI) =2.92 (1.52, 5.59)] more likely to discard colostrum compared to mothers who gave birth at health institution (Table 4).

**Table 4:** Binary and multivariable logistic regression analysis showing factors associated with colostrum avoidance among mothers of children aged less than 24 months in North Wollo zone, Northeastern Ethiopia, 2015

Variables	Colostrum avoidance n(%)	COR (95% CI)	AOR (95% CI)
Early initiation of breastfeeding			
Yes	50(8.3)	1	1
No	31(18.5)	2.49 (1.53,4.05)**	2.03(1.18,3.49)*
Prelacteal feeding			
Yes	29(34.9)	6.548 (3.84, 11.15)**	3.38(1.83,6.24) **
No	52(7.6)	1	1
Antenatal care checkup			
Yes	79(10.7)	1	1
No	15(31.9)	3.89 (2.02,7.50**	1.64(0.72,3.72)
Maternal awareness on advantages of colostrum			
Yes	55(10.9)	1	1
No	39(14.0)	1.33 (0.86,2.07)	1.34(0.81,2.24)
Sex of the index child			
Female	38(10.3)	1	1
Male	56(13.6)	1.37 (0.88,2.12)	1.348(0.81,2.25)
Marital status			
Living together	71(10.4)	1	1
Not living together	23(22.8)	2.53 (1.49,4.29)*	2.24(1.22,4.11)*
Parity			
1	56(15.5)	1	1
2–3	29(8.8)	0.53 (0.33,0.85)*	0.66(0.38,1.15)
≥ 4	9(9.9)	0.60 (0.29,1.26)	0.64(0.274,1.49)
Place of delivery			
Health institution	69(9.9)	1	1
Home	25(30.5)	4.01(2.36,6.83)**	2.92(1.52,5.59)*

Lemeshow goodness-of-fit = 0.142. *COR* Crude odds ratio, *AOR* Adjusted odds ratio *CI* Confidence interval. *significant at *p* < 0.05. **significant at *p* < 0.001

## Discussion

Proper breastfeeding practices are essential for the growth, development and survival of children. Despite this fact, colostrum is discarded in different parts of the globe since it is thought as yellowish-dirty milk [7, 13–17, 22]. This study showed that colostrum avoidance was practiced by 12.0% (95% CI: 10.00–14.00%) of mothers of children aged less than 24 months which is higher than the findings reported at Kersa district of Ethiopia (8.5%) [12]. Similar findings were reported at Raya Kobo (13.5%) [7] and Arbaminch zuria (11.0%) [23]. However, the prevalence of colostrum avoidance in this study was lower compared to the national prevalence in Ethiopia (39.8%) [20], Bahir Dar city (16.7%) [24], Jimma Arjo district (27.5%) [6], Goba district (35%) [13] and India (15.2%) [25]. This difference might be due to socio-cultural factors that affect colostrum feeding.

Mothers who initiated breastfeeding after 1 h of delivery were more likely to discard colostrum than mothers who initiated breastfeeding within 1 h after delivery. This is in line with the findings at Raya Kobo district [7]. This could be because of the fact that those mothers who initiated breastfeeding lately would have more time for infant feeding malpractices like colostrum avoidance. The reverse might be also correct. When mothers tend to discard colostrum, they might take more time to discard colostrum and initiate breastfeeding later [11].

Prelacteal feeding is positively associated with colostrum avoidance. In the current study, mothers who practiced prelacteal feeding were more likely to throw away colostrum compared to their counterparts. In Egypt mothers considered colostrum as bad to their baby and majority of mothers introduced prelacteal feeds in the first feed [26]. In support of this idea in Ethiopia, newborns fed on prelacteal foods before breastfeeding initiation. This is because prelacteal foods were believed to decrease infant mortality and morbidity [7, 27]. But colostrum is discarded since it was considered as expired yellowish-dirty milk that could cause abdominal cramp [7, 23, 27].

Maternal marital status is associated with colostrum avoidance in the current study area. Compared to mothers who live with their husband, mothers who live without their husband were more likely to discard colostrum. Similarly household head mothers were more likely to discard colostrum compared with mothers who were not household heads [7]. This might be due to the fact that mothers who live without their husband might lack the helpful advices on the importance of colostrum feeding.

Mothers who delivered the index child at home were 2.9 times more likely to discard colostrum compared to mothers who gave birth at health institution. Similarly in northeastern Ethiopia mothers who delivered the index child at home were 2.6 times more likely to discard colostrums as compared with mothers who gave birth at health institutions [7]. This could be grandmothers [7, 23, 28] and traditional birth attendants are the influential individuals for colostrum avoidance [7, 23]. Therefore, delivering at home might prevent women from immediate colostrum feeding in the face of these potentially influential individuals. On the other hand mothers who delivered in health institution might have knowledge provided by health workers which can improve attitude of mothers towards colostrum feeding.

## Conclusion

Colostrum was discarded by 12.0% of mothers of children aged less than 24 months. Colostrum avoidance was more common among mothers who initiated breastfeeding lately, practiced prelacteal feeding, live without their husband and delivered the index child at home. However, the foundation of any nutrition package for the prevention of childhood malnutrition is the promotion of an optimal breastfeeding practices, including colostrum feeding, in the community. Therefore, promoting institutional delivery, early initiation of breastfeeding and creating awareness on the dangers of prelacteal feeding and the advantages of colostrum feeding are recommended interventions to reduce colostrum avoidance.
